# Inequalities in Nutrition between Cambodian Women over the Last 15 Years (2000–2014)

**DOI:** 10.3390/nu8040224

**Published:** 2016-04-19

**Authors:** Valérie Greffeuille, Prak Sophonneary, Arnaud Laillou, Ludovic Gauthier, Rathmony Hong, Rathavuth Hong, Etienne Poirot, Marjoleine Dijkhuizen, Frank Wieringa, Jacques Berger

**Affiliations:** 1 JRU NUTRIPASS IRD-SupAgro-UM, Montpellier 34000, France; gauthier.ludo@hotmail.fr (L.G.); franck.wieringa@ird.fr (F.W.); jacques.berger@ird.fr (J.B.); 2 National Nutrition Program, Maternal and Child Health Center, No 31A, Rue de France (St. 47), Phnom Penh 12202, Cambodia; sophonprak@gmail.com; 3 UNICEF, Maternal, Newborn and Child Health and Nutrition section, no11 street 75, Phnom Penh 12202, Cambodia; alaillou@unicef.org (A.L.); rhong@unicef.org (R.H.); epoirot@unicef.org (E.P.); 4 ICF International, 530 Gaither Road, Suite 500, Rockville, MD 20850, USA; rathavuth.hong@icfi.com; 5 Department of Human nutrition, Copenhagen University, Rolighedsvej 26, 1958 Frederiksberg, Denmark; madijkhuizen@gmail.com

**Keywords:** women, underweight, overweight, anemia, Cambodia, Southeast Asia

## Abstract

This study aimed to describe the trends over four nationally representative Demographic Health Surveys (2000, 2005, 2010, and 2014) of the nutritional status of women of reproductive age in Cambodia and to assess the main factors of inequality with regards to nutrition. The prevalence of undernutrition and over-nutrition evolved in opposite trends from 2000 to 2014, with a significant decrease in underweight and a significant increase in overweight women. This results in a shift, with overweight prevalence in women being higher than underweight for the first time in 2014. Anemia was constantly high and still affected 45% of women in 2014. Multivariate analysis showed that age, wealth index, maternal education, number of children, year of survey, and anemia were contributing factors for being underweight. Being overweight was positively related to increase in age, wealth index, number of children, and year of survey; and negatively related to anemia and increase in education level. The risk of anemia was higher in the poorest households and for less-educated women and women living in rural areas. Consequently, policies should target the most vulnerable women, especially the youngest, and support integrated interventions in the health, social, and agriculture sectors to reduce inequalities in nutrition between women.

## 1. Introduction 

Women of reproductive age and children are the most at-risk populations for malnutrition, which covers under-nutrition, micronutrient deficiencies, overweight, and obesity. The prevalence of underweight women has decreased all over the world in the past decade but remains a concern in Cambodia where, in 2010, 19% of women had a body mass index (BMI) <18.5kg/m^2^ [1]. Anemia, defined as low hemoglobin concentration, is one of the main micronutrient deficiencies that affect women. Anemia decreases work capacity and causes fatigue in adults. If occurring during pregnancy, it increases the risks of adverse pregnancy outcomes [2]. For the year 2011, it was estimated that 29% of all women of reproductive age had anemia [3].

All forms of malnutrition negatively affect the health status of women and, in addition, during pregnancy, the development and health of their offspring.

Under-nutrition in women may have serious consequences for both maternal and child morbidity and mortality. Low maternal weight is associated with an increased risk of maternal mortality [4,5], increased risk of preterm birth, and neonates that are small for their gestational age [6,7,8,9]. Over-nutrition also has deleterious consequences for the health of both women and children [9,10,11]. Being overweight or obese is associated with increased risk of weakened health and increased relative risk of non-communicable diseases [10]. These forms of malnutrition are associated with adverse pregnancy outcomes, including: gestational mellitus diabetes, induction of labor, post-partum hemorrhage, and intra-uterine death [9,11]. An increased risk of high body weight of offspring in both infancy and childhood has been reported for women who are overweight at conception [9,10].

Although many countries have made considerable efforts to improve maternal and child health and nutrition, progress remains to be made as inequalities in nutrition persist [12,13,14,15,16,17]. Consequently, the aim of this study was to (1) describe the trends across four DHS surveys (2000, 2005, 2010, 2014) of women’s nutritional status; (2) assess the main factors of inequalities (age, education, living area, and wealth) and their evolution; and (3) assess the association between women’s anthropometry and a series of inequality factors.

## 2. Data and Methodologies

### 2.1. Data Sources

This study used data from the Cambodia Demographic Health Surveys (CDHS) conducted in 2000, 2005, 2010, and 2014 (Macro international Inc., Opinion Research Corporation (ORC Macro), Caverlton, Maryland, USA) on women of reproductive age from 15 to 49 years old [1,18,19,20]. The DHS surveys collect information on household demographic and socioeconomic characteristics and on child anthropometry, child feeding practices, and child health in a nationally representative sample. The surveys were based on stratified samples selected at two stages, and each reporting domain was separated into rural and urban areas.

### 2.2. Indicators Used

Anthropometric measurements were collected from samples of women aged from 15 to 49, excluding pregnant women and women who had given birth in the two months preceding the survey. Women were measured to the nearest 1 mm and weighed to the nearest 100 g. A body mass index (BMI) of <18.5 kg/m^2^ was used to define underweight, and overweight was defined using both the international cutoff and the specific cutoff for Asian populations of >25 kg/m^2^ and >23 kg/m^2^, respectively [21].

Anemia was defined as a hemoglobin concentration below 110 g/L for pregnant women and below 120 g/L for non-pregnant women. The concentration of hemoglobin was corrected by subtracting 3 g/L in women who reported that they were regular smokers [22]. The concentration of hemoglobin was measured on a subsample of the population of women aged from 15 to 49 years old.

The analysis of inequality was carried out between subgroups of the population constructed according to different socio-demographic factors usually implicated in inequalities and defined by DHS survey. Age was analyzed in three categories: younger than 20 years old, from 20 to 34 years old, and from 35 to 49 years old. The education level was categorized as “none” for women who never attended school, “primary” for women who have attended some primary school without necessarily having completed it, and “secondary+” for women who have attended some secondary school or more. The living area was categorized as urban or rural. The household wealth index was constructed using principal component analysis, as described before [23], with assets that were consistently available over time. This included data concerning accessibility and type of water, sanitation facility, materials used for housing construction, type of fuel used for cooking, and ownership of selected assets such like a radio, television, refrigerator, *etc.* Wealth quintiles were used to analyze the data (poorest, poorer, middle, richer, and richest categories). The reference groups were, respectively: the youngest women (<20 years), the women who did not attend school, the women living in rural areas, and the poorest women.

### 2.3. Statistical Analysis

Analysis was performed using STATA v11, using the STATA’s *svyset* function to integrate the complex sampling design of DHS surveys (stratification, clustering, and sampling).

Standard errors were estimated using the Taylor series linearization method, which incorporates sampling weights and used variance formulas appropriate for the DHS sample design. *Z*-tests on weighted percentages made it possible to compare the results between surveys.

Analysis of the inequalities were carried out using logistic regressions to determine whether, for each time frame, the absolute differences in prevalence (absolute inequality) observed between subgroups were statistically significant. When statistically significant (*p* < 0.05), the difference in prevalence between subgroups of population was reported in bold in tables. Additionally, the relative inequality between subgroups was assessed by calculating the Odds Ratio (OR) between subgroups. When statistically significant (*p* < 0.05), the OR between subgroups of population was reported in bold in tables. Analysis of the trends of nutritional status over time was carried out using logistic regression to determine whether, for each subgroup, the absolute differences in prevalence observed between each time frame were statistically significant. When statistically significant (*p* < 0.05), the difference of prevalence between the survey in subgroups of population is reported in bold. Both differences in prevalence and Odds Ratios (OR) between the extreme subgroups (indicated in parenthesis in the tables) with the associated standard errors were reported.

Multivariate logistic regressions were carried out to model the nutritional status of women as a function of their socioeconomic characteristics over the past 14 years. The covariates used to build the model were: age (in four categories: <20 years, 20 to 26 years, 27 to 34 years, and 35 to 49 years); education level (none, primary, secondary level), living area (urban/rural), wealth quintile (poorest, poorer, middle, richer, and richest categories) and number of children (three categories: no child, one to three children, more than three children). Variables in the model were selected through a backward stepwise conditional approach. Any variables that were not significant in the model (*p* > 0.05) were excluded except for age, which was kept in the model even if not significant. Analysis included all women from whom hemoglobin was collected and who were measured and weighed, except those flagged with extreme BMI for underweight and overweight analysis. Both the p-value and the non-normalized β-coefficients were reported. β-coefficients indicated the direction of relationship between independent and dependent variables. Negative values of the β-coefficient indicated a negative contribution of the explanatory variable when it changes from reference category to the next category.

## 3. Results

The mean height, weight, BMI, and hemoglobin concentration in women increased significantly between 2000 and 2014 (Table 1) overall and in both urban and rural living areas, respectively*.* Over time, the prevalence of women with no education decreased consistently from 28.2% in 2000 to 12.8% in 2014. The prevalence of women attaining the secondary level of education rose consistently and was multiplied by 2.3 between 2000 and 2014.

**Figure 1:**
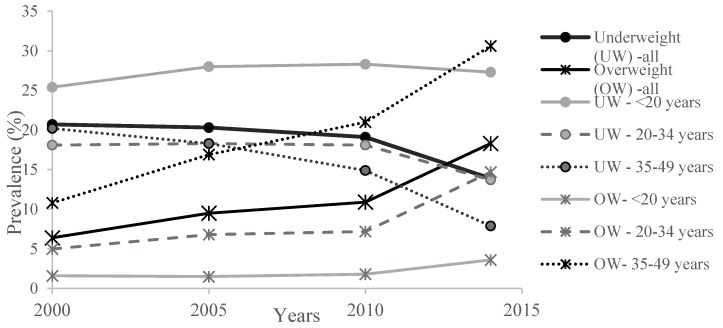
Percentage of underweight and overweight women by age groups and year of survey.

The prevalence of underweight women decreased consistently and significantly over time, particularly between 2010 and 2014 (Table 2 and Figure 1). In all surveys, underweight affected significantly more of the youngest women under 20 years of age. Notably, while the prevalence of being underweight in younger women did not change significantly over the study period, it decreased significantly in older women. The risk of being thin was significantly higher in the youngest women compared to the oldest women in all surveys, and increased from 1.2 to 3.5 from 2000 to 2014. Prevalence of being underweight was also linked to the wealth status of women and was significantly higher in the women belonging to the lowest wealth quintiles in all surveys. Although women in rural areas were more likely to be underweight than those in urban areas until 2010, in 2014 there was no significant difference in the prevalence of underweight women between rural and urban living areas. 

The prevalence of being overweight (OW) increased consistently and significantly from 2000 to 2014, especially between 2010 and 2014 when the OW prevalence increased from 10.9% to 18.3% (Table 3 and Figure 1). In all surveys, OW prevalence was linked to the age and was significantly higher in older women than in the youngest. The OR of being OW in older women ranged from 7.3 to 14.3 depending on the survey. The increase in OW prevalence over years was particularly important in the group of older women as it was almost multiplied by 3 (Figure 1). While not significantly related to education level in the 2000 and 2005 surveys, the risk of being OW was higher in women with no education compared with those with a secondary level or higher in the 2010 and 2014 surveys. OW prevalence and risk were higher in women living in urban areas, despite the fact that OW prevalence increased similarly and significantly over time in both areas. In all four surveys, the prevalence of being OW was linked to the wealth category, with the risk of being OW being significantly higher in the wealthiest category. The prevalence of being OW using a specific cutoff for Asian populations (BMI ≥ 23 kg/m^2^, [21]) was higher and increased from 15.7% in 2000 to 33.3% in 2014 (Table S1, supplementary material). The prevalence and risks of being OW showed the same relationships with age, education level, living area, and wealth index as for OW calculated with international cutoffs.

The prevalence of anemia was high in all surveys and, after a significant decrease between 2000 and 2005, remained stable at approximately 45% (Table 4). The prevalence of anemia was not related to age, except in 2005 where the prevalence was significantly higher in older women. In all surveys, the prevalence of anemia and the risk of being anemic were significantly lower in women with a secondary level of education compared to women with no education. Anemia was linked to the wealth quintiles with approximately half the risk of being anemic in the richest category compared to the poorest category of the wealth index.

The multivariate analysis of the four DHS surveys showed that the significant factors contributing to being underweight were: age, wealth index, maternal education, number of children, year of survey, and anemia (Table 5). Analysis showed that the risk of being underweight decreased as age increased, wealth index increased, maternal education decreased, number of children increased, and year of survey increased. Being underweight was also linked to anemia, the risk being higher in women with anemia. In contrast, being overweight was positively linked to an increase in age group, wealth index score, and number of children; and negatively linked to anemia and an increase in education level. The risk of anemia decreased as age increased, education level increased, and year of survey increased. The risk of anemia is also higher in people living in rural areas. This analysis also illustrated the decrease in the risks of being underweight and anemic from 2000 to 2014 and the increase in risk of being overweight.

## 4. Discussion

As can be expected in a country in transition, the prevalence of undernutrition and overnutrition in Cambodian women evolved in opposite ways between 2000 and 2014, with a significant overall decrease in the prevalence of underweight women and a significant increase in the prevalence of overweight women [24]. In 2014, for the first time, the prevalence of overweight women exceeded the prevalence of underweight women.

In each of the four Cambodian surveys (2000–2014), being underweight was of major concern, mostly in the youngest category of women, *i.e.*, those under 20, and this inequality has increased over the last 14 years. The overall prevalence of being underweight in women under 20 was 27% in 2014, a figure that has not changed since 2000, whereas the prevalence of being underweight decreased by two thirds in older women. These results point to the special attention that needs to be paid to adolescents girls, especially in developing countries such as Cambodia, where a large proportion of young women marry at an early age and become pregnant during the last years of adolescence. Attention should thus focus on this particularly vulnerable population since being underweight during pregnancy has been shown to be linked with low birth weight and poor pregnancy outcomes [5,6,7,8].

Overall, the associations between wealth status and being underweight are consistent with other studies showing that the poorest individuals are the most at risk of underweight [12,13,17]. In Cambodia, both food insecurity and low dietary diversity scores were found to be determinants of maternal underweight cases [25] and probably contributed to the underweight cases in the four national surveys. This inequality of the relationship between being underweight and wealth status has decreased over the last 14 years, and although a statistical significant difference still existed in the prevalence of being underweight of 2.4% between the poorest and the richest quintile in 2014, it is questionable whether this difference was of importance in the context of public health.

From 2000 to 2010, being underweight was more prevalent in rural areas in Cambodia, as found in the national representative survey carried out in 2010 in Vietnam, a neighboring country that has long borders with Cambodia [26]. However, in the most recent survey (2014) there were no differences in the prevalence of being underweight between urban and rural areas. Multivariate analysis of the four surveys showed that education level was significantly linked to the prevalence of being underweight, with the most educated women being the most at risk of being underweight, especially in 2014, as shown in univariate analysis. Age, wealth index, number of children, and anemia were also identified as contributing factors to being underweight in the last 14 years. This suggests that while food security might have increased since 2000, thereby reducing the overall prevalence of being underweight among youngest women, especially in urban areas but probably also in women of high socioeconomic status, the desire to remain slim might offset this trend.

Using international cutoffs, the prevalence of being overweight followed the opposite trend to the prevalence of being underweight and increased slightly from 2000 to 2010 and more rapidly thereafter, reaching almost one fifth of all women and one third of women over 35 years of age in 2014. Similar increases in being overweight have been observed in several countries, especially in Southeast Asia, with an estimated global prevalence of being overweight of 28.3% in 2013 for women aged 20 years and older [27]. In that study, the authors estimated that the prevalence of being overweight for women aged 20 or more in Cambodia was 18.3%, ranking the country alongside Vietnam and Timor Leste with the lowest prevalence of being overweight in women in the region. Using cutoffs recommended for Asian populations, the prevalence of being overweight approximately doubled in each survey (33.4% in 2014), starting to be as high as the figures for being underweight in 2005, and higher thereafter.

Regarding multivariate analysis of the four DHS surveys, age, wealth status, education level, number of children, and anemia contributed significantly to being overweight. In 2014, the risk of being overweight was especially high, in fact 11.7 times higher, in older women compared to the youngest. These results are consistent with international findings that show that being overweight particularly affects older women [28], and women belonging to the richest class [29]. Being overweight is not significantly associated with living area when considering the four DHS surveys. However, multivariate analysis run only for the 2014 survey showed that the risk of being overweight is higher in urban areas (*p*-value = 0.049, data not shown). This inequality between rural and urban areas appeared in 2010 and persisted in 2014. This inequality towards being overweight has already been reported for other countries [27,29]. 

Even if being overweight mostly affected the richest population, the inequality between wealth groups has tended to narrow since 2010, when a more rapid increase was observed in the prevalence of being overweight in the poorest households. Indeed, since 2000, the prevalence of being overweight in the poorest group has increased five-fold, whereas the prevalence of being overweight in the wealthiest group has ‘only’ doubled. Similar tendencies were already reported a few years ago in many countries [15,29]. Our findings on the relationships between being overweight and socioeconomic indicators did not differ when being overweight was defined with Asian cutoffs. Jones-Smith *et al.* (2012) reported a greater growth rate of being overweight in lower wealth class and educational groups in several countries [29]. This may be due to several factors, among them an increased access to energy-rich foods and changes in physical activities as being overweight is primarily driven by imbalanced diets and more sedentary lifestyles [30,31]. 

While overweight prevalence was not related to the level of education in the 2000 and 2005 surveys, the risk of being overweight was higher in women with no education in the 2010 and 2014 surveys, with a shift of the most-at-risk group from the highest to lowest educational groups. This relationship has been demonstrated in several reviews concluding that, in low-income countries, the best educated women are less likely to be overweight or obese [32,33]. This direct relationship between low education level and being overweight suggests an increasing awareness and knowledge of the risks associated with being overweight and obese in the most educated groups, as suggested in a recent review [33].

Anemia was a severe public health problem [34] in all four Cambodian surveys with over 40% of the women being affected in all the surveys. Significant associations were found with education level, living area, and wealth in each survey, as well as in the multivariate analysis performed on the pooled data of the four surveys. In contrast, a recent, smaller study in women in rural Cambodia showed that hemoglobin concentration was associated with age, pre-menopausal status, and the quantity of meat consumed, but not with socioeconomic status [35].

Anemia is of multifactorial origin but both nutritional and non-nutritional factors contribute. From the four Cambodian surveys, it appears that the prevalence of anemia cannot be reduced below 40%, even in women of the highest wealth group or with the highest education. This underlines an important role of non-nutritional causes in the etiology of anemia in Cambodia. Indeed, in Cambodia, it is estimated that almost half of the population is affected by some sort of hemoglobin disorder, hemoglobin E being the most prevalent [36,37]. Such disorders result in anemia due to impaired hemoglobin synthesis and a recent study suggested that these disorders are more closely associated with hemoglobin concentrations in rural women in Cambodia than iron deficiency [36]. Yet it is interesting to note that, according to the multivariate analysis, over 14 years, anemia has been positively associated with being underweight and negatively associated with being overweight, reinforcing the hypothesis that, although a significant part of the anemia is due to non-nutritional causes, the deficiency of iron and other micronutrients, most likely due to a low quality of diet in underweight women, still plays a role in the overall high prevalence of anemia in Cambodia. There is thus still a need for policies to increase food and nutritional security and diet diversity, probably with increased consumption of animal and/or micronutrient-fortified foods.

Although the inflammatory status of women was not evaluated in three of the four surveys, we cannot exclude the possibility that it played a role in the burden of anemia as well as parasitic infection that affected, in 2014, 18.4% of mothers aged 15–49 years who have had a child since 2009 [20]. A better understanding of all the causes and their contribution to anemia would help to design appropriate intervention strategies and policies, and inform policymakers on appropriate targets to reduce the prevalence of anemia in women.

Overall, these results show that Cambodia is facing the double burden of malnutrition in women, with a global tendency to an increase in being overweight and obese while undernutrition remains a public health problem in younger age groups. The socioeconomic characteristics of women have also changed over time, with a constant decrease in the number of women with no education, still estimated at 13% in 2014, and a global improvement in the socioeconomic context and in the situation of women over the last 14 years. Indeed, during the last 14 years, the Gross National Income (GNI) per capita increased from $300 in 2000 to $1020 in 2014 and, in 2014, the annual percentage growth rate of the gross domestic product (GDP) was 7.1% [38]. Since 2000, Cambodia has made important efforts to develop and strengthen the maternal and child health system [12]. These actions probably contributed to the global decrease of undernutrition in the country. Other direct determinants of malnutrition such as food security should also be considered to support the improvements. Actions to improve the availability, accessibility, and affordability of nutrient-rich food would contribute to the reduction of both undernutrition and micronutrient deficiencies. Finally, the extension of the drinking water network and sanitation systems would also contribute to the improvement of the nutritional status of the Cambodian population. Consequently, Cambodian policymakers should develop several complementary actions: firstly, actions against undernutrition, including both nutrition-sensitive and nutrition-specific interventions targeting first the poorest, the youngest, and the women living in rural areas; secondly, concerning overweight cases, policies should include a prevention campaign to explain the behavioral factors favoring being overweight and the consequences on health; finally, an appropriate food and agriculture policy should be implemented to favor the availability of the micronutrient-rich food rather than carbohydrate- or lipid-dense foods.

This paper is not without some limitations. Although the data analyzed here were nationally representative, the results of our analysis should be interpreted with caution, keeping in mind that numerous drivers of inequality exist, such as economic development, climate change, urbanization, conflicts, *etc.*, depending on time or area considered. Nevertheless, this study focused on some of the factors of inequality that may exist and these factors are among those most often reported in the analysis of inequality toward nutritional status. Moreover, the analysis presented here comprised both absolute and relative measures of inequalities as recommended by Harper *et al.* [39] to produce a complete and objective description of the data.

## 5. Conclusions

The socioeconomic condition of women in Cambodia has improved significantly over the last 15 years, and today Cambodia is on the brink of becoming a middle-income country. Over this period of time, the nutritional status of women has changed significantly, with a decrease in the prevalence of being underweight combined with an increase in the prevalence of being overweight. Cambodia is thus now facing a double burden of malnutrition in women and has to define and implement appropriate strategies to improve the nutritional status of women, in particular that of adolescent girls and young women. The prevalence of anemia has not changed significantly in the last decade, and non-nutritional causes are likely to be the main contributor to the high prevalence of anemia in Cambodia. Realistic targets for anemia prevention programs need to be set, but to do so, a better understanding of the etiology of anemia in Cambodia is needed. Over the last 14 years, and even in 2014, socioeconomic characteristics such as place of residence, wealth status, education level, and age give women multiple risk factors for being malnourished. Complementary actions specific to each socioeconomic subgroup of women should be implemented, taking into account recent trends in the prevalence of malnutrition inside each of these subgroups, with a particular focus on the youngest women. Nutrition-specific and nutrition-sensitive actions could be designed to reduce the number of underweight women. Furthermore, policies focusing on the behavioral causes of being overweight would help to reduce this problem. Finally, interventions favoring the production of micronutrient-rich foods should be developed in both the agriculture and the agri-food sectors.

## Figures and Tables

**Table 1:** Characteristics of the women included in DHS surveys in 2000, 2005, 2010, and 2014.

		2000			2005			2010			2014			Year Effect
		*n*	% or Mean	Sd Err *	*n*	% or Mean	Sd Err	*n*	% or Mean	Sd Err	*N*	% or Mean	Sd Err	*p Value*
Age (mean)		15,351	**30.1**	0.1	16,823	**30.2**	0.1	18,754	**30.2**	0.1	17,577	**30.7**	0.1	<0.001
Age groups	<20 years	3564	**23.6**	0.4	3646	**21.4**	0.4	3915	**19.9**	0.4	3006	**16.5**	0.3	<0.001
	20 to 34 years	6340	**41.0**	0.5	7159	**42.7**	0.5	8559	**45.8**	0.5	8899	**50.6**	0. 6	<0.001
	35 to 49 years	5447	**35.4**	0.4	6018	**35.9**	0.4	6280	**34.3**	0.4	5672	**32.9**	0.5	<0.001
Height (m)		7499	**1.52**	0.001	8357	**1.52**	0.001	9332	**1.53**	0.001	11,484	**1.53**	0.001	<0.001
Weight (kg)		7505	**48.3**	0.04	8364	**48.8**	0.05	9336	**49.3**	0.05	11,487	**51.6**	0.05	<0.001
BMI		7467	**20.6**	0.05	8350	**21.0**	0.04	9327	**21.1**	0.04	11,479	**22.0**	0.04	<0.001
Hemoglobin content (g/dL)		3666	**11.5**	0.07	8180	**11.8**	0.03	9224	**12.0**	0.03	11,411	**12.1**	0.05	<0.001
Living area	Urban	2656	**17.5**	0.6	4152	**17.7**	0.4	6077	**21.0**	0.6	5667	**18.5**	0.7	0.030
	Rural	12,901	**82.5**	0.6	12,671	**82.3**	0.4	12,677	**79.0**	0.6	11,910	**81.5**	0.7	0.030
Education level	None	4849	**28.2**	0.9	3772	**19.4**	0.7	3203	**15.8**	0.6	2233	**12.8**	0.6	<0.001
	Primary	8182	**54.6**	0.7	9131	**55.8**	0.7	8796	**49.4**	0.7	7825	**47.1**	0.7	<0.001
	Secondary	2320	**17.2**	0.7	3920	**24.7**	0.8	9755	**34.7**	0.9	7519	**40.1**	0.8	<0.001
Mother with a childbirth during the last two years		9784	**62.2**	0.5	10,685	**62.6**	0.5	11,783	**63.7**	0.5	11,660	**67.6**	0.5	<0.001
Mean number of children/by women		15,351	**2.2**	0.0	16,823	**2.0**	0.0	18,754	**1.8**	0.0	17,577	**1.8**	0.0	<0.001

* Standard Error.

**Table 2:** Prevalence of underweight in women in the four surveys according to their social characteristics.

	Prevalence % (Standard Error)				Trends over Time ^δ^		
Characteristic	2000	2005	2010	2014	2000–2014	2005–2014	2010–2014
AGE							
<20	25.4 (1.26)	28.0 (1.42)	28.3 (1.38)	27.3 (1.29)	1.9	−0.7	−1	
20–34	18.1 (0.88)	18.3 (0.87)	18.1 (0.67)	13.7 (0.61)	−4.4 *	−4.6 *	−4.4 *
35–49	20.2 (0.87)	18.3 (0.87)	14.9 (0.85)	7.9 (0.58)	−12.3 *	−10.4 *	−7 *
Absolute difference (Old-Young)	−5.2 *	−9.7 *	−13.4 *	−19.4 *	−14.2 *	−9.7 *	−6 *
OR (Old:Young)	0.74 * (0.06)	0.57 * (0.05)	0.44 * (0.04)	0.23 * (0.02)			
EDUCATION							
None	22.0 (1.19)	19.1 (1.22)	18.6 (1.15)	10.8 (1.09)	−11.2 *	−8.3 *	−7.8 *
Primary	19.7 (0.77)	20.4 (0.82)	17.6 (0.79)	12.3 (0.56)	−7.4 *	−8.1 *	−5.3 *
Secondary+	21.5 (1.45)	21.1 (1.19)	21.3 (0.88)	16.9 (0.77)	−4.6 *	−4.2 *	−4.4 *
Absolute difference (Second.-None)	−0.5	2	2.7	6.1 *	6.6 *	4.1 *	3.4 *
OR (Second.:None)	0.97 (0.11)	1.14 (0.12)	1.18 (0.11)	1.67 * (0.21)			
RESIDENCE							
Urban	16.1 (1.35)	17.3 (1.21)	16.8 (0.9)	13.4 (0.77)	−2.7	−3.9 *	−3.4 *
Rural	21.6 (0.67)	21 (0.68)	19.7 (0.59)	14 (0.5)	−7.6 *	−7 *	−5.7 *
Absolute difference (Urban-Rural)	5.5 *	3.7 *	2.9 *	0.6	−4.9 *	−3.1	−2.3
OR (Urban:Rural)	0.69 * (0.07)	0.78 * (0.07)	0.82 * (0.06)	0.95 (0.07)			
WEALTH QUINTILE							
Poorest	24.5 (1.4)	24.1 (1.38)	22.4 (1.28)	14.9 (0.97)	−9.6 *	−9.2 *	−7.5 *
Poorer	21.4 (1.53)	22 (1.21)	20.1 (1.24)	14.4 (0.97)	−7 *	−7.6 *	−5.7 *
Middle	20.8 (1.24)	22.7 (1.34)	18.7 (1.43)	15.3 (1.02)	−5.5 *	−7.4 *	−3.4 *
Richer	20.6 (1.4)	18.2 (1.23)	18.3 (1.14)	13.2 (0.98)	−7.4 *	−5 *	−5.1 *
Richest	16.8 (1.1)	16.4 (1.29)	16.6 (0.81)	12.3 (0.76)	−4.5 *	−4.1 *	−4.3 *
Absolute difference (Richest-Poorest)	−7.7 *	−7.7 *	−5.8 *	−2.6 *	5.1	5.1	3.2
OR (Richest:Poorest)	0.62 * (0.07)	0.62 * (0.07)	0.68 * (0.07)	0.81 * (0.08)			
Total	20.7 (0.60)	20.3 (0.60)	19.1 (0.50)	13.9 (0.42)	−6.8 *	−6.4 *	−5.2 *
*N*	7467	8350	9327	11,479			

* *p*-value < 0.05: indicates significant differences over time or between subgroups. δ trends over time correspond to absolute difference in prevalence between each period of survey.

**Table 3:** Prevalence of overweight in women in the four surveys according to their social characteristics.

	Prevalence % (Standard Error)				Trends over Time ^δ^		
Characteristic	2000	2005	2010	2014	2000–2014	2005–2014	2010–2014
AGE							
<20	1.6 (0.36)	1.5 (0.36)	1.8 (0.39)	3.6 (0.56)	2.0 *	2.1 *	1.8 *
20–34	5.0 (0.52)	6.8 (0.57)	7.2 (0.52)	14.7 (0.61)	9.7 *	7.9 *	7.5 *
35–49	10.8 (0.79)	16.9 (0.96)	21.0 (1.12)	30.6 (1.04)	19.8 *	13.7 *	9.6 *
Absolute difference (Old-Young)	9.2 *	15.4 *	19.2 *	27 *	17.8 *	11.6	7.8
OR (Old:Young)	7.28 * (1.70)	13.37 * (3.13)	14.28 * (3.11)	11.74 * (1.94)			
EDUCATION							
None	5.5 (0.59)	9.4 (1.02)	13.1 (1.29)	22.8 (1.51)	17.3 *	13.4 *	9.7 *
Primary	6.7 (0.50)	10.0 (0.67)	11.8 (0.72)	20.6 (0.75)	13.9 *	10.6 *	8.8 *
Secondary+	6.5 (0.88)	8.6 (0.76)	8.7 (0.69)	14.2 (0.64)	7.7 *	5.6 *	5.5 *
Absolute difference (Second.-None)	1	−0.8	−4.4 *	−8.6 *	−9.6 *	−7.8 *	−4.2
OR (Second.:None)	1.18 (0.22)	0.90 (0.13)	0.63 * (0.08)	0.56 * (0.05)			
RESIDENCE							
Urban	9.6 (1.17)	13.4 (1.09)	15.7 (1.3)	22.9 (0.9)	13.3 *	9.5 *	7.2 *
Rural	5.7 (0.4)	8.7 (0.57)	9.6 (0.58)	17.3 (0.61)	11.6 *	8.6 *	7.7 *
Absolute difference (Urban-Rural)	3.9 *	4.7 *	6.1 *	5.6 *	1.7	0.9	−0.5
OR (Urban:Rural)	1.76 * (0.27)	1.63 * (0.19)	1.75 * (0.21)	1.43 * (0.09)			
WEALTH QUINTILE							
Poorest	2.4 (0.57)	4.1 (0.62)	4.8 (0.73)	12 (0.99)	9.6 *	7.9 *	7.2 *
Poorer	4.7 (0.65)	4.2 (0.61)	8.6 (0.86)	17 (1.11)	12.3 *	12.8 *	8.4 *
Middle	5.3 (0.75)	6.7 (0.88)	9.8 (0.95)	17.5 (1.15)	12.2 *	10.8 *	7.7 *
Richer	6.5 (0.80)	11.9 (0.98)	13.2 (1.14)	19.8 (1.08)	13.3 *	7.9 *	6.6 *
Richest	11.7 (1.06)	17.6 (1.30)	16.3 (1.26)	23.5 (0.93)	11.8 *	5.9 *	7.2 *
Absolute difference (Richest-Poorest)	9.3 *	13.5 *	11.5 *	11.5 *	2.2 *	−2 *	0
OR (Richest:Poorest)	5.3 * (1.39)	5.02 * (0.91)	3.88 * (0.69)	2.24 * (0.24)			
Total	6.4 (0.39)	9.5 (0.50)	10.9 (0.53)	18.3 (0.52)	11.9 *	8.8 *	7.4 *
N	7467	8350	9327	11479			

* *p*-value < 0.05: indicates significant differences over time or between subgroups; ^δ^ trends over time correspond to absolute differences in prevalence between each period of survey.

**Table 4:** Prevalence of anemia ^1^ in women according to their social characteristics.

	Prevalence % (Standard Error)				Trends over Time *^δ^*		
Characteristic	2000	2005	2010	2014	2000–2014	2005–2014	2010–2014
AGE							
<20	57.3 (2.27)	44.7 (1.61)	46.8 (1.57)	49.2 (1.4)	−8.1 *	4.5 *	2.4
20–34	55.3 (1.47)	43.2 (1.11)	41.9 (1.07)	43.1 (0.83)	−12.2 *	**−**0.1	1.2
35–49	59.9 (1.64)	50.2 (1.08)	45.7 (1.13)	48.1 (1.07)	−11.8 *	**−**2.1	2.4
Absolute difference (Old-Young)	2.6	5.5 *	−1.1	−1.1	−3.7	**−6.6**	0.0
OR (Old:Young)	1.11 (0.12)	1.24 * (0.09)	0.96 (0.07)	0.96 (0.07)			
EDUCATION							
None	62.2 (2.02)	52.8 (1.66)	49.1 (1.86)	49.1 (1.73)	−13.1 *	−3.7	0
Primary	57.4 (1.44)	46.9 (0.9)	46 (0.98)	47.8 (0.86)	−9.6 *	0.9	1.8
Secondary+	49.3 (2.58)	38.5 (1.3)	39.3 (1.14)	42.1 (0.84)	−7.2 *	3.6 *	2.8 *
Absolute difference (Second.-None)	−12.9 *	−14.3 *	−9.8 *	−7.0 *	5.9	7.3 *	2.8
OR (Second.:None)	**0.59** * (0.08)	**0.56** * (0.05)	**0.67** * (0.06)	0.75 * (0.06)			
RESIDENCE							
Urban	51 (2.83)	36 (1.69)	34.4 (1.31)	39.4 (1.01)	−11.6	3.4	5
Rural	58.8 (1.14)	48.2 (0.83)	46.8 (0.88)	47.2 (0.71)	−11.6	−1	0.4
Diff (Urban-Rural)	−7.8 *	−12.2 *	−12.4 *	−7.8 *	0	4.4 *	4.6 *
OR (Urban:Rural)	0.73 * (0.09)	0.60 * (0.05)	0.60 * (0.04)	0.73 * (0.04)			
WEALTH QUINTILE							
Poorest	64.4 (2.07)	56.9 (1.63)	52.5 (1.76)	53.5 (1.52)	−10.9 *	−3.4	1
Poorer	60.1 (2.33)	49.6 (1.54)	48.1 (1.49)	48.5 (1.36)	−11.6 *	−1.1	0.4
Middle	58.6 (2.34)	48.8 (1.5)	46.1 (1.76)	45.7 (1.39)	−12.9 *	−3.1	−0.4
Richer	57.5 (2.55)	46.6 (1.56)	43.2 (1.62)	43.9 (1.29)	−13.6 *	−2.7	0.7
Richest	47.6 (2.29)	32.6 (1.44)	33.9 (1.22)	39.1 (1.18)	−8.5 *	6.5 *	5.2 *
Absolute difference (Richest-Poorest)	−16.8 *	−24.3 *	−18.6 *	−14.4 *	2.4	9.9 *	4.2
OR (Richest-Poorest)	0.50 * (0.06)	0.37 * (0.03)	0.46 * (0.04)	0.55 * (0.04)			
Total	57.4 (1.07)	46.0 (0.75)	44.2 (0.75)	45.7 (0.61)	−11.7 *	**−**0.3	1.5
N	3666	8180	9224	11,411			

* *p*-value < 0.05: indicates significant differences over time or between subgroups; ^1^ Cutoffs adjusted for smoking; pregnant <110 g/L and non-pregnant <120 g/L); ^δ^ trends over time correspond to absolute differences in prevalence between each period of survey.

**Table 5:** *P*-value and regression coefficients of factors contributing to underweight, overweight, and anemia prevalence in Cambodian women in the four DHS surveys.

	Underweight (*N* = 30,654)		Overweight (*N* = 30,654)		Anemia (*N* = 32,470)	
	*p*-Value	β-Coefficient * (95% CI)	*p*-Value	β-Coefficient * (95% CI)	*p*-Value	β-Coefficient * (95% CI)
Increase in age category **	<0.001	−0.21 (−0.26 −0.17)	<0.001	0.71 (0.65 0.77)	ns	-
Increase in wealth index	<0.001	−0.12 (−0.15 −0.09)	<0.001	0.36 (0.32 0.40)	<0.001	−0.13 (−0.16 −0.11)
Increase in maternal education category	0.007	0.09 (0.02 0.15)	<0.001	−0.18 (−0.26 −0.11)	<0.001	−0.08 (−0.13 −0.03)
Living in urban area (ref: rural area)					<0.001	-0.15 (−0.23 0.10)
Increase in number of children	<0.001	−0.17 (−0.25 −0.10)	<0.001	0.24 (0.16 0.32)	-	-
Increase in year of survey	<0.001	−0.04 (−0.05 −0.03)	<0.001	0.09 (0.08 0.10)	<0.001	−0.02 (−0.03 −0.01)
Having anemia (ref: no anemia)	<0.001	0.22 (0.15 0.30)	<0.001	−0.49 (−0.58 −0.39)		

* Negative values of the coefficient indicate a negative contribution of the explanatory variable (column) when it changes from one category to the next category; ** Age was included in the model even if non–significant.

## Supplementary Materials

The following are available online at http://www.mdpi.com/2072-6643/8/4/224/s1, Table S1: Prevalence of overweight (BMI ≥ 23 kg/m^2^) in women, according to their social characteristics.

## References

[1] (2011). Cambodia Demographic Health Survey, 2010.

[2] WHO (2015). The Global Prevalence of Anaemia in 2011.

[3] Stevens G.A., Finucane M.M., De-Regil L.M., Paciorek C.J., Flaxman S.R., Branca F., Peña-Rosas J.P., Bhutta Z.A., Ezzati M. (2011). Global, regional, and national trends in haemoglobin concentration and prevalence of total and severe anaemia in children and pregnant and non-pregnant women for 1995–2011: A systematic analysis of population-representative data. Lancet Glob. Health.

[4] Parul C., Katz J., Wu L., Kimbrough-Pradhan E., Khatry S.K., LeClerq S.C., West K.P. (2008). Risk factors for pregnancy-related mortality: A prospective study in rural nepal. Public Health.

[5] WHO (1995). Maternal anthropometry and pregnancy outcomes. A who collaborative study. Bull. World Health Organ..

[6] Fujiwara K., Aoki S., Kurasawa K., Okuda M., Takahashi T., Hirahara F. (2014). Associations of maternal pre-pregnancy underweight with small-for-gestational-age and spontaneous preterm birth, and optimal gestational weight gain in japanese women. J. Obstet. Gynaecol. Res..

[7] Han Z., Mulla S., Beyene J., Liao G., McDonald S.D. (2011). Maternal underweight and the risk of preterm birth and low birth weight: A systematic review and meta-analyses. Int. J. Epidemiol..

[8] Black R.E., Allen L.H., Bhutta Z.A., Caulfield L.E., de Onis M., Ezzati M., Mathers C., Rivera J. (2008). Maternal and child undernutrition: Global and regional exposures and health consequences. Lancet (Lond. UK).

[9] Yu Z., Han S., Zhu J., Sun X., Ji C., Guo X. (2013). Pre-pregnancy body mass index in relation to infant birth weight and offspring overweight/obesity: A systematic review and meta-analysis. PLoS ONE.

[10] Ryan D. (2007). Obesity in women: A life cycle of medical risk. Int. J. Obes..

[11] Sebire N.J., Jolly M., Harris J.P., Wadsworth J., Joffe M., Beard R.W., Regan L., Robinson S. (2001). Maternal obesity and pregnancy outcome: A study of 287,213 pregnancies in london. Int. J. Obes. Relat. Metab. Disord..

[12] Cesare M.D., Bhatti Z., Soofi S.B., Fortunato L., Ezzati M., Bhutta Z.A. (2015). Geographical and socioeconomic inequalities in women and children’s nutritional status in pakistan in 2011: An analysis of data from a nationally representative survey. Lancet Glob. Health.

[13] Kamal S.M., Hassan C.H., Alam G.M. (2015). Dual burden of underweight and overweight among women in bangladesh: Patterns, prevalence, and sociodemographic correlates. J. Health Popul. Nutr..

[14] Mendez M.A., Monteiro C.A., Popkin B.M. (2005). Overweight exceeds underweight among women in most developing countries. Am. J. Clin. Nutr..

[15] Monteiro C.A., Conde W.L., Lu B., Popkin B.M. (2004). Obesity and inequities in health in the developing world. Int. J. Obes. Relat. Metab. Disord.

[16] Simkhada B., Teijlingen E.R., Porter M., Simkhada P. (2008). Factors affecting the utilization of antenatal care in developing countries: Systematic review of the literature. J. Adv. Nurs..

[17] Tebekaw Y., Teller C., Colon-Ramos U. (2014). The burden of underweight and overweight among women in addis ababa, ethiopia. BMC Public Health.

[18] (2000). Cambodia Demographic Health Survey, 2000.

[19] (2005). Cambodia Demographic Health Survey, 2005.

[20] (2015). Cambodia Demographic Health Survey 2014.

[21] consultation W.E. (2004). Appropriate body-mass index for asian populations and its implications for policy and intervention strategies. Lancet.

[22] UNICEF, UNU, WHO (2001). Iron Deficiency Anaemia Assessment, Prevention, and Control—A Guide for Programme Managers.

[23] Filmer D., Pritchett L.H. (2001). Estimating wealth effects without expenditure data—Or tears: An application to educational enrollments in states of india. Demography.

[24] Jaacks L.M., Slining M.M., Popkin B.M. (2015). Recent underweight and overweight trends by rural-urban residence among women in low- and middle-income countries. J. Nutr..

[25] McDonald C.M., McLean J., Kroeun H., Talukder A., Lynd L.D., Green T.J. (2014). Household food insecurity and dietary diversity as correlates of maternal and child undernutrition in rural cambodia. Eur. J. Clin. Nutr..

[26] Laillou A., Pham T.V., Tran N.T., Le H.T., Wieringa F., Rohner F., Fortin S., Le M.B., Tran do T., Moench-Pfanner R. (2012). Micronutrient deficits are still public health issues among women and young children in vietnam. PLoS ONE.

[27] Ng M., Fleming T., Robinson M., Thomson B., Graetz N., Margono C., Mullany E.C., Biryukov S., Abbafati C., Abera S.F. (2014). Global, regional, and national prevalence of overweight and obesity in children and adults during 1980–2013: A systematic analysis for the global burden of disease study 2013. Lancet (Lond. UK).

[28] Jones-Smith J.C., Gordon-Larsen P., Siddiqi A., Popkin B.M. (2011). Cross-national comparisons of time trends in overweight inequality by socioeconomic status among women using repeated cross-sectional surveys from 37 developing countries, 1989–2007. Am. J. Epidemiol..

[29] Jones-Smith J.C., Gordon-Larsen P., Siddiqi A., Popkin B.M. (2012). Is the burden of overweight shifting to the poor across the globe? Time trends among women in 39 low- and middle-income countries (1991–2008). Int. J. Obes. (2005).

[30] Mistry S.K., Puthussery S. (2015). Risk factors of overweight and obesity in childhood and adolescence in south asian countries: A systematic review of the evidence. Public Health.

[31] Popkin B.M., Adair L.S., Ng S.W. (2012). Global nutrition transition and the pandemic of obesity in developing countries. Nutr. Rev..

[32] Cohen A.K., Rai M., Rehkopf D.H., Abrams B. (2013). Educational attainment and obesity: A systematic review. Obes. Rev. Off. J. Int. Assoc. Study Obes..

[33] Devaux M., Sassi F., Church J., Cecchini M., Borgonovi F. (2011). Exploring the relationship between education and obesity. OECD J. Econ. Stud..

[34] WHO (2011). Haemoglobin Concentrations for the Diagnosis of Anemia and Assessment of Severity.; World Health Organization.

[35] Charles C.V., Dewey C.E., Hall A., Hak C., Channary S., Summerlee A.J. (2015). Anemia in cambodia: A cross-sectional study of anemia, socioeconomic status and other associated risk factors in rural women. Asia Pac. J. Clin. Nutr..

[36] Karakochuk C.D., Whitfield K.C., Barr S.I., Lamers Y., Devlin A.M., Vercauteren S.M., Kroeun H., Talukder A., McLean J., Green T.J. (2015). Genetic hemoglobin disorders rather than iron deficiency are a major predictor of hemoglobin concentration in women of reproductive age in rural prey veng, cambodia. J. Nutr..

[37] Weatherall D.J., Clegg J.B. (2001). Inherited haemoglobin disorders: An increasing global health problem. Bull. World Health Org..

[38] World Development Indicators 2015. http://data.worldbank.org/country/cambodia.

[39] Harper S., King N.B., Meersman S.C., Reichman M.E., Breen N., Lynch J. (2010). Implicit value judgments in the measurement of health inequalities. Milbank Q..

