# Supplementary material for: Inequalities in Nutrition between Cambodian Women over the Last 15 Years (2000–2014)
Source: Nutrients. 2016 Apr 19;8(4):224. doi: 10.3390/nu8040224 (PMC4848692; doi:10.3390/nu8040224)
Supplement: Supplementary file 1 [file nutrients-08-00224-s001.docx]

Supplementary Materials: Inequalities in Nutrition between Cambodian Women over the Last 15 Years (2000–2014)

Valérie Greffeuille, Prak Sophonneary, Arnaud Laillou, Ludovic Gauthier, Rathmony Hong, Rathavuth Hong, Etienne Poirot, Marjoleine Dijkhuizen,
Frank Wieringa and Jacques Berger

**Table S1.** Prevalence of overweight (BMI ≥ 23 kg/m^2^) in women, according to their social characteristics.

|  | **% (Sd.Err)** | | | | **Comparison** | | |
| --- | --- | --- | --- | --- | --- | --- | --- |
| **Characteristic** | **2000** | **2005** | **2010** | **2014** | **2014–2000** | **2014–2005** | **2014–2010** |
| **AGE AT CHILD BIRTH** | |  |  |  |  |  |  |
| <20 year | 6.8 (0.7) | 7.7 (0.8) | 7.8 (0.8) | 11.0 (0.9) | **4.2 *** | **3.3 *** | **3.2 *** |
| 20–34 year | 14.9 (0.9) | 17.6 (0.9) | 17.1 (0.8) | 29.5 (0.9) | **14.6 *** | **11.9 *** | **12.4 *** |
| 35–49 year | 22.4 (1.0) | 31.2 (1.2) | 36.8 (1.4) | 49.8 (1.1) | **27.4 *** | **18.6 *** | **13.0 *** |
| Diff (Old-Young) | **15.6 *** | **23.5 *** | **29.0 *** | **38.8 *** | **23.2 *** | 15.3 | 9.8 |
| O-R (Old:Young) | **3.94 *** (0.48) | **5.46 *** (0.67) | **6.85 *** (0.84) | **8.02 *** (0.78) |  |  |  |
| EDUCATION |  |  |  |  |  |  |  |
| None | 14.7 (1.0) | 20.0 (1.3) | 24.9 (1.5) | 39.1 (1.8) | **24.4 *** | **19.1 *** | **14.2 *** |
| Primary | 16.4 (0.8) | 21.5 (0.9) | 23.9 (0.9) | 37.1 (0.9) | **20.7 *** | **15.6 *** | **13.2 *** |
| Secondary+ | 15.1 (1.3) | 19.4 (1.1) | 18.3 (1.0) | 27.2 (1.0) | **12.1 *** | **7.8 *** | **8.9 *** |
| Diff (Second.-None) | 0.4 | −0.6 | **−6.6 *** | **−11.9 *** | **−12.3 *** | **−11.3 *** | −5.3 |
| O-R (Second.: None) | 1.03 (0.13) | 0.96 (0.10) | **0.67** * (0.07) | **0.58** * (0.05) |  |  |  |
| RESIDENCE |  |  |  |  |  |  |  |
| Urban | 22.5 (1.7) | 27.3 (1.4) | 28.1 (1.7) | 36.9 (0.9) | **14.4 *** | **9.6 *** | **8.8 *** |
| Rural | 14.3 (0.6) | 19.2 (0.7) | 20.5 (0.7) | 32.6 (0.8) | **18.3 *** | **13.4 *** | **12.1 *** |
| Diff (Urban-Rural) | **8.2 *** | **8.1 *** | **7.6 *** | **4.3 *** | **−3.9 *** | **−3.8 *** | **−3.3 *** |
| O-R (Urban : Rural) | **1.73 *** (0.19) | **1.58 *** (0.13) | **1.52 *** (0.14) | **1.21 *** (0.06) |  |  |  |
| WEALTH QUANTILE | | |  |  |  |  |  |
| Poorest | 9.2 | 11.2 | 15.5 | 26.1 | **16.9 *** | **14.9 *** | **10.6 *** |
| Poorer | 12.7 | 13.9 | 18 | 32.5 | **19.8 *** | **18.6 *** | **14.5 *** |
| Middle | 13.9 | 16.6 | 20.8 | 31.4 | **17.5 *** | **14.8 *** | **10.6 *** |
| Richer | 15.7 | 23.4 | 25.4 | 35.5 | **19.8 *** | **12.1 *** | **10.1 *** |
| Richest | 25 | 33.3 | 28.4 | 39.6 | **14.6 *** | **6.3 *** | **11.2 *** |
| Diff (Richest-Poorest) | **15.8 *** | **22.1 *** | **12.9 *** | **13.5 *** | −2.3 | −8.6 | 0.6 |
| O-R (Richest-Poorest) | **3.28 *** (0.45) | **3.95 *** (0.45) | **2.17 ***(0.23) | **1.86 *** (0.16) |  |  |  |
| Total | 15.7 (0.6) | 20.7 (0.66) | 22.1 (0.66) | 33.4 (0.68) | **17.7 *** | **12.7 *** | **11.3 *** |
